# Genetic diversity of the 2013–14 human isolates of influenza H7N9 in China

**DOI:** 10.1186/s12879-015-0829-8

**Published:** 2015-02-28

**Authors:** Amber Farooqui, Alberto J Leon, Linxi Huang, Suwu Wu, Yingmu Cai, Pengzhou Lin, Weihong Chen, Xibin Fang, Tiansheng Zeng, Yisu Liu, Li Zhang, Ting Su, Weibin Chen, Elodie Ghedin, Huachen Zhu, Yi Guan, David J Kelvin

**Affiliations:** Division of Immunology, International Institute of Infection and Immunity, Shantou University Medical College, 22 Xinling Road, Shantou, Guangdong 515041 China; Division of Experimental Therapeutics, University Health Network, Toronto, Ontario Canada; Intensive Care Unit, the First Affiliated Hospital of Shantou University Medical College, Shantou, Guangdong China; Intensive Care Unit, Shantou Central Hospital, Shantou, Guangdong China; Department of Laboratory Medicine, The First Affiliated Hospital of Shantou University Medical College, Shantou, Guangdong China; Department of Biology, Center for Genomics and Systems Biology, Global Institute of Public Health, New York University, New York, USA; Joint Influenza Research Centre (SUMC/HKU), Shantou University Medical College, Shantou, China; State Key Laboratory of Emerging Infectious Diseases/Centre of Influenza Research, School of Public Health, The University of Hong Kong, Hong Kong, SAR China

## Abstract

**Background:**

Influenza H7N9 has become an endemic pathogen in China where circulating virus is found extensively in wild birds and domestic poultry. Two epidemic waves of Human H7N9 infections have taken place in Eastern and South Central China during the years of 2013 and 2014. In this study, we report on the first four human cases of influenza H7N9 in Shantou, Guangdong province, which occurred during the second H7N9 wave, and the subsequent analysis of the viral isolates.

**Methods:**

Viral genomes were subjected to multisegment amplification and sequenced in an Illumina MiSeq. Later, phylogenetic analyses of influenza H7N9 viruses were performed to establish the evolutionary context of the disease in humans.

**Results:**

The sequences of the isolates from Shantou have closer evolutionary proximity to the predominant Eastern H7N9 cluster (similar to A/Shanghai/1/2013 (H7N9)) than to the Southern H7N9 cluster (similar to A/Guangdong/1/2013 (H7N9)).

**Conclusions:**

Two distinct phylogenetic groups of influenza H7N9 circulate currently in China and cause infections in humans as a consequence of cross-species spillover from the avian disease. The Eastern cluster, which includes the four isolates from Shantou, presents a wide geographic distribution and overlaps with the more restricted area of circulation of the Southern cluster. Continued monitoring of the avian disease is of critical importance to better understand and predict the epidemiological behaviour of the human cases.

**Electronic supplementary material:**

The online version of this article (doi:10.1186/s12879-015-0829-8) contains supplementary material, which is available to authorized users.

## Background

**Figure 1:**
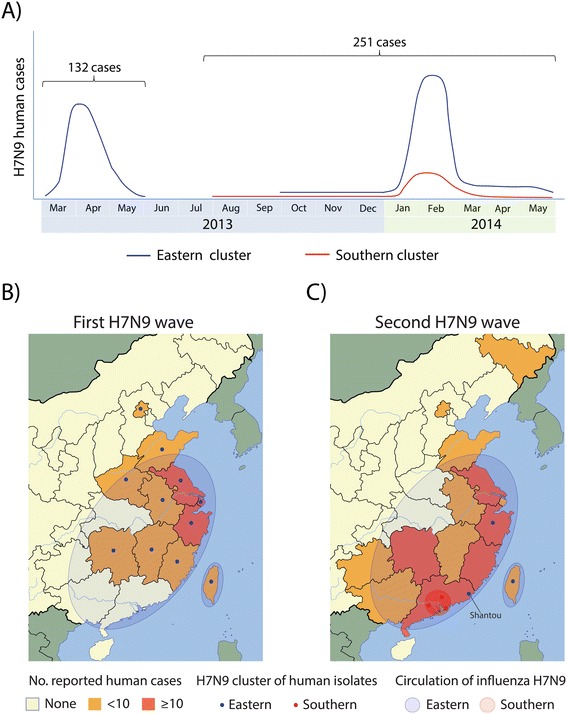
**Temporal, geographic and genetic distribution of human cases of influenza H7N9. A)** Evolution in the number of reported influenza H7N9 infections in humans caused by viruses from the Eastern cluster (similar to A/Shanghai/1/2013(H7N9) and the Southern cluster (similar to A/Guangdong/1/2013(H7N9)). **B)** and **C)** Geographic distribution of human cases during the first and second waves of the disease, respectively. The number of cases and their locations were obtained from the WHO case reports on influenza H7N9 (http://www.wpro.who.int/outbreaks_emergencies/H7N9.archive/en/). The genetic classification of the H7N9 clusters is based on the phylogenetic analysis of the viral isolates. Shaded areas represent the territories in which the presence of the virus has been detected by surveillance studies.

A novel influenza H7N9 virus (A/Shanghai/1/2013 (H7N9) and related isolates) emerged in China in March of 2013, causing 32 reported deaths by May 30 and a case fatality rate of 25% [1]. After the first series of human cases in Shanghai [2], the outbreak spread to multiple provinces of Eastern China although the majority of cases were detected in the eastern provinces of Jiangsu, Shanghai and Zhejiang (Figure 1A-B). This new virus originated as a result of gene reassortments from other circulating avian influenza viruses from the H7N7 and H7N9 subtypes [3]. Circulation of the new H7N9 virus in chickens and other avian species was reported in affected regions, and most human cases had a history of poultry contact [4-7]. This evidence suggests a zoonotic origin for the human H7N9 disease. Interestingly, viral surveillance revealed circulation of H7N9 in poultry markets in the southern province of Guangdong but without causing human disease [8], indicating that the presence of the H7N9 virus in a region did not systematically lead to human disease, or was possibly under-diagnosed.

A new variant of H7N9 (A/Guangdong/1/2013 (H7N9) and related isolates) was first detected in humans in August 2013 and caused a number of infections in humans in Guangdong and Hong Kong during the months following the initial outbreak (Figure 1C). This new variant was also the result of gene reassortment, with genes originating from avian influenza strains that were circulating in the South of China [8,9]. Later, a second wave of human cases of H7N9 (related to A/Shanghai/1/2013 (H7N9)) began after September 2013 and affected the Eastern and Southern provinces of China. Environmental surveillance data and epidemiological reports revealed that influenza H7N9 had achieved the status of a circulating strain among avian species and causing human disease as a consequence of sustained cross-species spillover [10].

Here we analyzed the genomic sequences of four influenza H7N9 viruses that were isolated in Shantou (Guangdong province, People’s Republic of China) during March and April of 2014. We also analyzed the genetic diversity of influenza H7N9 isolates that caused human disease.

## Methods

### Patients

Four patients with no apparent epidemiological relationship were admitted to the ICU unit of the Shantou Central Hospital and the ICU unit of the First Affiliated Hospital of Shantou University Medical College in Shantou (Guangdong province, People’s Republic of China) in March and April 2014. The patients presented influenza-like symptoms that included cough, fever, fatigue and severe pneumonia that developed into acute respiratory distress syndrome (ARDS). Two of the patients (#1 and #4) died due to irreversible deterioration of their clinical status, and the other two (#2 and #3) fully recovered. Upon admission, lung aspirates were collected through a suction catheter inserted into the endotracheal tube. A first analysis of the samples performed by China CDC confirmed the presence of influenza H7N9. A complete description of the clinical findings is to be published as part of a separate manuscript. This study was approved by the Ethics Committee of the Shantou Central Hospital and by the Ethics Committee of the First Affiliated Hospital of Shantou University Medical College. Each patient provided written informed consent.

### Virus isolation and sequencing

Lung aspirates were cleared by centrifugation and inoculated into chicken eggs for viral growth. Total RNA was extracted from 100 μl of allantoic fluid using Trizol LS (Life Technologies) according to the manufacturer’s protocol. Complete viral genomes were amplified by multisegment reverse-transcription PCR (M-RTPCR), a method that allows simultaneous amplification of all viral segments by using a pair of universal primers targeted against highly conserved flanking regions, as previously described [11]. Briefly, RNA was subjected to reverse transcription and PCR amplification with the SuperScript III One-Step RT-PCR System (Life Technologies), using the primers MBTuni12-ACGCGTGATCAGCAAAAGCAGG and MBTuni13-ACGCGTGATCAGTAGAAACAAGG and the following thermal cycler program: 42°C for 60 min and 94°C for 2 min; 5 cycles of 94°C for 30 sec, 45°C for 30 sec and 68°C for 3 min; 31 cycles of 94°C for 30 sec, 57°C for 30 sec and 68°C for 3 min. Sequencing libraries were prepared by using the Nextera XT DNA Sample Preparation kit (Illumina) according to the manufacturer’s protocol, and 150 bp paired-end sequencing was performed in a MiSeq instrument (Illumina). The resulting short reads were aligned with respect to the sequences of A/Shanghai/1/2013 (H7N9) by Bowtie2 (2.2.3) [12] and the consensus sequences were generated with SAMtools (1.0) [13], as previously described [14]. The sequence variants were called with VarScan (2.3.6) [15] and only considered if causing aminoacid substitution and with a frequency ≥2%.

### Phylogenetic analyses

To establish the evolutionary context of the isolates from Shantou, phylogenetic analyses with the published H7N9 nucleotide sequences were performed. The genomic sequences of H7N9 isolates were retrieved from in GISAID’s EpiFlu database as of December 31^st^ 2014. Isolates were grouped according to their collection date as follows: first wave (February 1^st^ to June 30^th^, 2013), and second wave (July 1^st^ 2013 to June 30^th^ 2014). Only those isolates for which all their eight genomic segments were available with at least 75% of coverage were included in the analyses. In total, the genomic sequences from 95 human isolates were used in the analysis, including the Shantou strains. Additionally, 6 avian isolates and 4 environmental isolates that are representative of the core genetic groups and of the different reassortant sub-groups were included in the analysis (Additional file 1: Table S1). MEGA6 (v6.0.6) [16] was used to infer the evolutionary history for each viral segment by using the Maximum Likelihood method based on the General Time Reversible Model [17] and using 1000 bootstrap replications.

## Results

### Sequencing of four influenza H7N9 isolates of human origin

In this study, four influenza H7N9 viruses that were isolated from patients with severe pneumonia in Shantou during March and April of 2014 were characterized at the genomic level. The clinical specimens collected from these patients were inoculated into embryonated eggs for viral expansion and the resulting isolates were named A/Shantou/1001/2014 (H7N9), A/Shantou/1002/2014 (H7N9), A/Shantou/1003/2014 (H7N9) and A/Shantou/1004/2014 (H7N9). After amplification of the viral genomes by M-RTPCR, the sequencing of the PCR products resulted in approximately 184,000, 248,000, 214,000 and 192,000 viral short-reads, respectively. The resulting consensus sequences presented complete coverage of the viral genomes and they were deposited on GISAID’s EpiFlu database (isolate identifiers EPI_ISL_162618, EPI_ISL_162619, EPI_ISL_162620 and EPI_ISL_162621, respectively).

### The isolates from Shantou belong to the Eastern cluster of influenza H7N9

**Figure 2:**
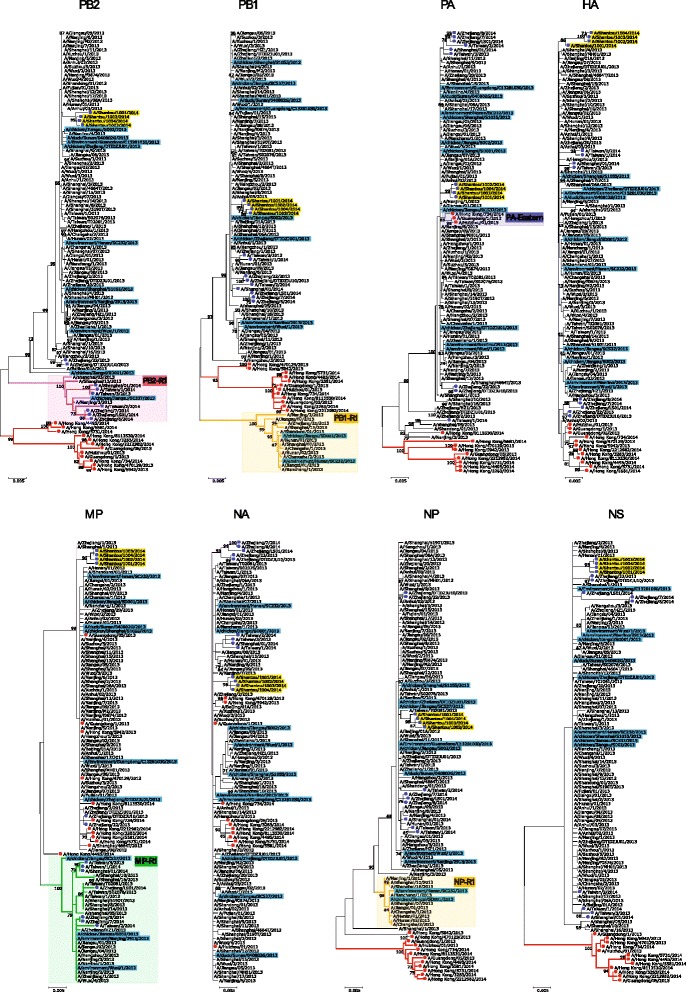
**Phylogenetic analysis of the viral segments of influenza H7N9 isolated from human patients.** Using the software program Mega 6.02, phylogenetic trees were inferred by using the Maximum Likelihood method based on the General Time Reversible Model with 1000 bootstrap replications. Bootstrap support values ≥70% are shown. Branch lengths are proportional to number of substitutions per site. Unmarked strain names: Eastern cluster from the first wave of the disease; blue circle: Eastern cluster from the second wave of the disease; red circle: Southern cluster from the second wave of the disease; Yellow highlight: viral isolates from Shantou; blue highlight: strain of avian or environmental origin.

**Figure 3:**
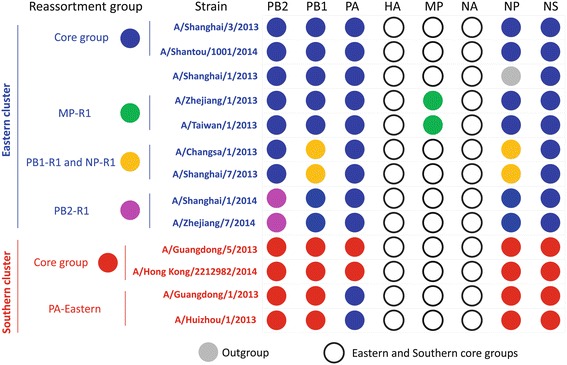
**Genetic make-up of the sub-groups of human isolates of influenza H7N9.** For each group, two representative strains were selected, and the phylogenetic origin of their genomic segments is shown by the colour code indicated in the panel.

Next, we sought to characterise the evolutionary context of the human disease. The phylogenetic analyses of the viral genes indicate that, despite the presence of reassortant groups, all the strains of the first wave of the disease share a common evolutionary history and they do not form apparent clusters by host species (Figure 2). A separate set of phylogenetic analyses that included the human isolates (n = 95), and the available avian (n = 44) and environmental (n = 21) isolates, also indicate that the strains are evenly distributed across reassortant groups regardless of their origin (data not shown). The strains of the second wave are divided between a first group whose gene sequences clustered together with the strains of the first wave, and a second group in which a majority of their genes (PB2, PB1, PA, NP and NS) formed separate clusters with an evolutionary history independent from the rest of the H7N9 strains. Therefore, we defined one first phylogenetic group of H7N9 strains named the Eastern cluster, formed by A/Shanghai/1/2013 and related strains in which the Shantou strains are included; we also defined a second group of H7N9 strains named the Southern cluster that includes A/Guangdong/1/2013 and related strains. Although both A/Shanghai/1/2013 and A/Guangdong/1/2013 carry reassortments that set them apart from their respective core groups (as shown in Figures 2 and 3), they are mentioned as representative of the Eastern and Southern clusters, respectively, for historical reasons and because of their higher relevance in the related literature.

The variability within the human isolates of the Eastern cluster is mainly caused by gene reassortments that led to the formation of groups of strains that include MP-R1, the double reassortment PB1-R1&NP-R1, and PB2-R1. Within the Southern cluster, a group of strains carry the PA gene of the Eastern cluster (PA-Eastern subgroup), possibly as a late re-introduction (Figures 2 and 3). The gene reassortments found in the human isolates of the Eastern cluster were also present in the avian and environmental isolates; unfortunately, no complete genomic sequences of avian or environmental samples belonging to the Southern cluster were available as of this writing and precluded us from performing a direct comparison of strains of different origin within this group.

### Aminoacid substitutions in the proteins of the Shantou strains

**Figure 4:**
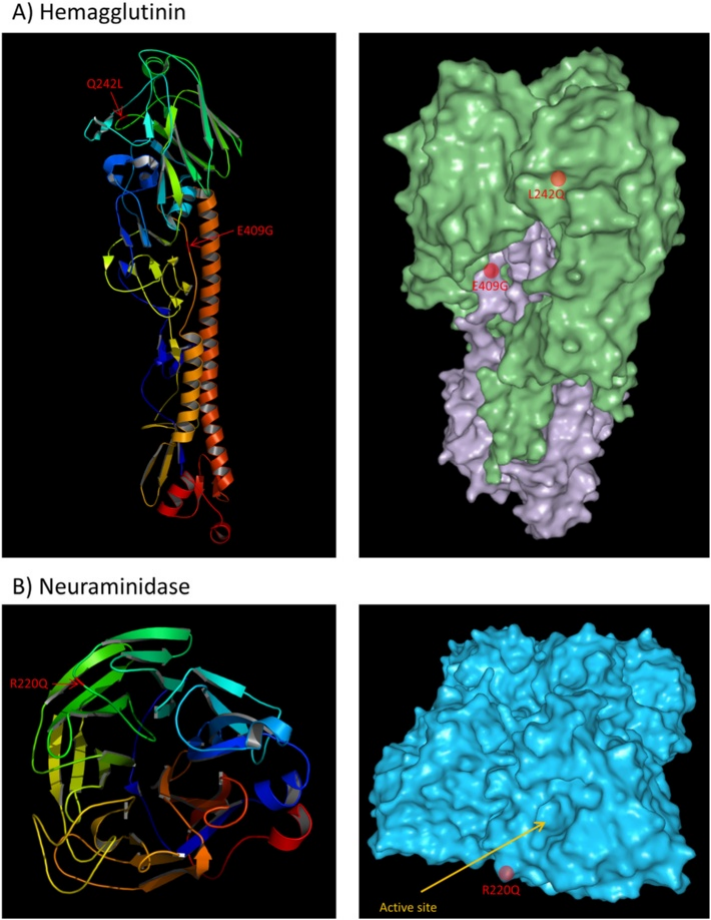
**Mapping of aminoacid substitutions found in the hemagglutinin A) and neuraminidase B) proteins that are present in the four Shantou isolates.** Ribbon diagrams were generated with Pymol (1.7.1.13) (http://www.pymol.org) and the 3D protein structures with RCSB’s Protein Workshop (http://www.rcsb.org) using PDB accessions 4KOL (HA) and 4MWL (NA).

At the aminoacid level, several novel substitutions were present in all four Shantou strains (Additional file 2: Table S2): PA (P271S and N321C), HA (E409G [H3 numbering]), M2 (V27I), NA (S78N and R220Q [both in N2 numbering]) and NS1 (S48I and A82P). Mapping the aminoacid substitutions to the 3D structures of the HA and NA genes (PDB accessions 4KOL and 4MWL, respectively) showed that HA-E409G belongs to the stalk domain of the HA2 protein, NA-S78N belongs to the stalk domain of the NA protein, and NA-R220Q is situated in an area of the NA protein surface not related to the active site (Figure 4).

A number of substitutions were detectable only in part of the short-read sequences and coexisted with the reference version; this is an indication of the presence of viral quasi-species carrying different versions of the protein (Additional file 2: Table S2). None of the aminoacid substitutions present in the Shantou strains matched the characteristic traits of the Southern cluster, ruling out the presence of those variant genes even at low concentration.

Nonetheless, the pathogenic implications of these aminoacid substitutions, if any, are presently unknown and would require further investigation.

## Discussion

Although it was shown that H7N9 virus has the capacity to adapt to mammalian hosts [18,19], for mammal-to-mammal transmission [20] and limited human-to-human transmission [7], there is, to date, little supporting evidence that any mammal-adapted strain has achieved sustained circulation. The fact that no species-specific clusters are detected in the phylogenetic analyses supports the idea that the isolates from human cases have arisen directly from the pool of H7N9 avian viruses that are circulating in each location. Genetic tuning of the virus through the incorporation of specific nucleotide substitutions and gene reassortments are responsible for modulating the viral fitness, level of pathogenicity, and transmissibility on different host species [9,21,22]. It is likely that the spectrum of circulating avian H7N9 strains is broader than the one observed in the human isolates due to the presence of sub-groups with lower capacity for causing human infections.

Newly emerged influenza H7N9 has been circulating among avian species in different provinces of China since early 2013 and causing severe infections in humans. The four human cases of influenza H7N9 from Shantou were severe infections that caused death in two of the patients. The isolated viruses belong to the Eastern cluster, which is the predominant phylogenetic group among H7N9 viruses with wide distribution across multiple provinces of China (Figure 1B-C). Phylogenetic analyses show that the Shantou isolates derive from a common ancestor without having undergone gene reassortments or major evolutionary changes due to antigenic drifting. These results indicate that these four viruses, which were isolated within the short period of just two months, all derive from the same local pool of influenza H7N9 strains that were circulating among avian species at that time.

Human infections with viruses from the Southern cluster started during the second wave of the disease [10]. The phylogenetic analyses of their segments reveal that the NP and NS genes [8], as well as the PB2, PB1 and PA genes, are part of a separate lineage, whereas their HA, NA and MP genes have the same phylogenetic history as the Eastern cluster (Figure 2). Surveillance studies performed in isolates from avian and environmental samples, and from human cases in the Guangdong province, showed sustained co-circulation of strains from the Eastern and Southern clusters in some areas [10]. Although gene reassortments between these two groups of viruses are possible, as shown by the incorporation of the PA-Eastern gene in several strains of the Southern cluster (Figures 2 and 3), both clusters have remained largely independent despite being co-circulating in those some areas. Nonetheless, the full extent of the geographical and host species distribution of the different H7N9 sub-groups will be established more precisely through additional monitoring of avian influenza.

## Conclusions

So far, circulation of viruses from both the Eastern and Southern clusters of influenza H7N9 is restricted to avian hosts. We found that the genetic differences between these two clusters are concentrated in the internal genes, which may be a consequence of distinct profiles of viral fitness and transmission capacity on different avian species. Therefore, the geographical distribution of the H7N9 clusters may be a direct consequence of the geographical distribution of their preferred hosts. Continued monitoring efforts of the avian disease and host specificity studies are necessary to further understand the avian disease and its impact on the human population.

## Additional files

Additional file 1: Table S1. List of influenza isolates include in the study. Additional file 2: Table S2. Frequency of nucleotide substitutions in the H7N9 Shantou isolates.

## Abbreviations

ARDS: Acute respiratory disease syndrome
HA: Hemagglutinin
MP: Matrix protein
NA: Neuraminidase
NP: Nucleoprotein
NS1: Non-structural 1 protein
PA: Polymerase acidic
PB1: Polymerase basic 1
PB2: Polymerase basic 2
